# The tradition algorithm approach underestimates the prevalence of serodiagnosis of syphilis in HIV-infected individuals

**DOI:** 10.1371/journal.pntd.0005758

**Published:** 2017-07-20

**Authors:** Bin Chen, Xiuming Peng, Tiansheng Xie, Changzhong Jin, Fumin Liu, Nanping Wu

**Affiliations:** State Key Laboratory for Diagnosis and Treatment of Infectious Diseases, Collaborative Innovation Center for Diagnosis and Treatment of Infectious Diseases, the First Affiliated Hospital, School of Medicine, Zhejiang University, Hangzhou, China; University of Connecticut Health Center, UNITED STATES

## Abstract

Currently, there are three algorithms for screening of syphilis: traditional algorithm, reverse algorithm and European Centre for Disease Prevention and Control (ECDC) algorithm. To date, there is not a generally recognized diagnostic algorithm. When syphilis meets HIV, the situation is even more complex. To evaluate their screening performance and impact on the seroprevalence of syphilis in HIV-infected individuals, we conducted a cross-sectional study included 865 serum samples from HIV-infected patients in a tertiary hospital. Every sample (one per patient) was tested with toluidine red unheated serum test (TRUST), *T*. *pallidum* particle agglutination assay (TPPA), and *Treponema pallidum enzyme immunoassay* (TP-EIA) according to the manufacturer’s instructions. The results of syphilis serological testing were interpreted following different algorithms respectively. We directly compared the traditional syphilis screening algorithm with the reverse syphilis screening algorithm in this unique population. The reverse algorithm achieved remarkable higher seroprevalence of syphilis than the traditional algorithm (24.9% vs. 14.2%, *p* < 0.0001). Compared to the reverse algorithm, the traditional algorithm also had a missed serodiagnosis rate of 42.8%. The total percentages of agreement and corresponding kappa values of tradition and ECDC algorithm compared with those of reverse algorithm were as follows: 89.4%,0.668; 99.8%, 0.994. There was a very good strength of agreement between the reverse and the ECDC algorithm. Our results supported the reverse (or ECDC) algorithm in screening of syphilis in HIV-infected populations. In addition, our study demonstrated that screening of HIV-populations using different algorithms may result in a statistically different seroprevalence of syphilis.

## Author summary

Syphilis remains a worldwide public health concern as there has been a global increase in the incidence of syphilis. Serologic tests are still considered the mainstay of syphilis diagnosis. Currently, there are three algorithms for screening of syphilis- traditional algorithm, reverse algorithm and European Centre for Disease Prevention and Control (ECDC) algorithm. But there is no uniform screening method for syphilis. Different surveys use different screening algorithm. Will the different screening algorithm influence the seroprevalence of serodiagnosis of syphilis in this unique population? For those infected with both syphilis and HIV, the situation is even more complex. To the best of our knowledge, no studies have analyzed the different algorithms for detecting syphilis in HIV-positive people. Therefore, we compared the results of the three syphilis screening algorithms in an attempt to evaluate their screening performance in this unique population. Our results supported the reverse (or ECDC) algorithm in screening of syphilis in HIV-infected populations. In addition, our study demonstrated that the tradition algorithm approach underestimates the prevalence of serodiagnosis of syphilis in HIV-infected individuals.

## Introduction

Syphilis is an ancient human disease caused by *Treponema pallidum*, which is mostly transmitted by sex activity. It remains a worldwide public health concern as there has been a global increase in the incidence of syphilis, especially among men who have sex with men (MSM). MSMs are a unique population that experience disproportionately high rates of HIV infection. Although clinical profiling of symptoms is important, serologic tests are still considered the mainstay of syphilis diagnosis. Serological tests for syphilis can be categorized into two types: the non-treponemal tests (NTT) such as rapid plasma reagin (RPR), toluidine red unheated serum test (TRUST), and Venereal Disease Research Laboratory (VDRL) tests. Other treponemal tests (TT) include the *T*. *pallidum* particle agglutination assay (TPPA), *T*. *pallidum* hemagglutination assay (TPHA), treponemal ELISA, and chemiluminescence methodologies[1].

Currently, there are three algorithms for screening of syphilis. First, the traditional screening algorithm commences with a non-treponemal assay followed by a confirmation with a treponemal test. Second, the reverse algorithm starts with a treponemal assay, and a reactive treponemal screening assay is followed by a quantitative non-treponemal assay. Third, the European Centre for Disease Prevention and Control (ECDC) algorithm-a modified reverse algorithm: a reactive treponemal screening test is followed by a second (and different) treponemal test but is not accompanied by a non-treponemal test[2]. All testing algorithms possess certain advantages and limitations. Consequently, there is no generally recognized diagnostic algorithm[3]. For those infected with both syphilis and HIV, the situation is even more complex[4]. For example, unusual serologic responses such as the prozone and sreofast phenomenon have been observed in HIV-infected individuals[5].

To the best of our knowledge, no studies have analyzed the different algorithms for detecting syphilis in HIV-positive people. Therefore, this study aimed to compare the results of three syphilis screening algorithms in an attempt to evaluate their screening performance in this unique population. Moreover, we examined whether the different screening algorithms significantly influenced the seroprevalence of syphilis in HIV-positive patients.

## Materials and methods

### Study design and specimens

We conducted a cross-sectional study to assess the impact of different syphilis screening algorithms in a HIV-positive population. Sample size was estimated to be 677 using $\text{N}={\text{Z}}_{\alpha }^{2}\text{P}\left(1-\text{P}\right)⁄\delta^{2}$, assuming 19.8% syphilis prevalence in HIV infected patients[6], with 3% precision and 95% level of confidence. We collected a convenience sample of discarded serum specimens from HIV patients undergoing serologic evaluation for HIV virus load in The First Affiliated Hospital, Medical College of Zhejiang University. The patients’ HIV infection status was confirmed by the detection of HIV antibodies in blood using enzyme-linked immunosorbent assay (ELISA) and western blot analysis. The following data abstracted from the hospital electronic medical record: age, sex, racial and ethnic identity, the route of HIV transmission, and the stage of AIDS (the name were anonymized in the supporting information).

### Ethics statement

This study was approved by the Institutional Ethics Committee of The First Affiliated Hospital, Medical College of Zhejiang University and complied with the Declaration of Helsinki guidelines.

### Serological testing

TRUST (Rongsheng Biotech Co., Ltd, Shanghai, China) was used as the non-treponemal test, and TPPA (Fujirebio INC, Tokyo, Japan) and TP-EIA (Wantai Biological Pharmacy Enterprise Co., Ltd, Beijing, China) were used as the treponemal tests. Every sample (one per patient) was tested by TRUST, TPPA, and TP-EIA simultaneously. All testing was performed according to the manufacturer’s instruction. The performing assay technician was unaware of the results of other testing, and all the results were reported independently.

### Serodiagnosis of syphilis

**Fig 1:**
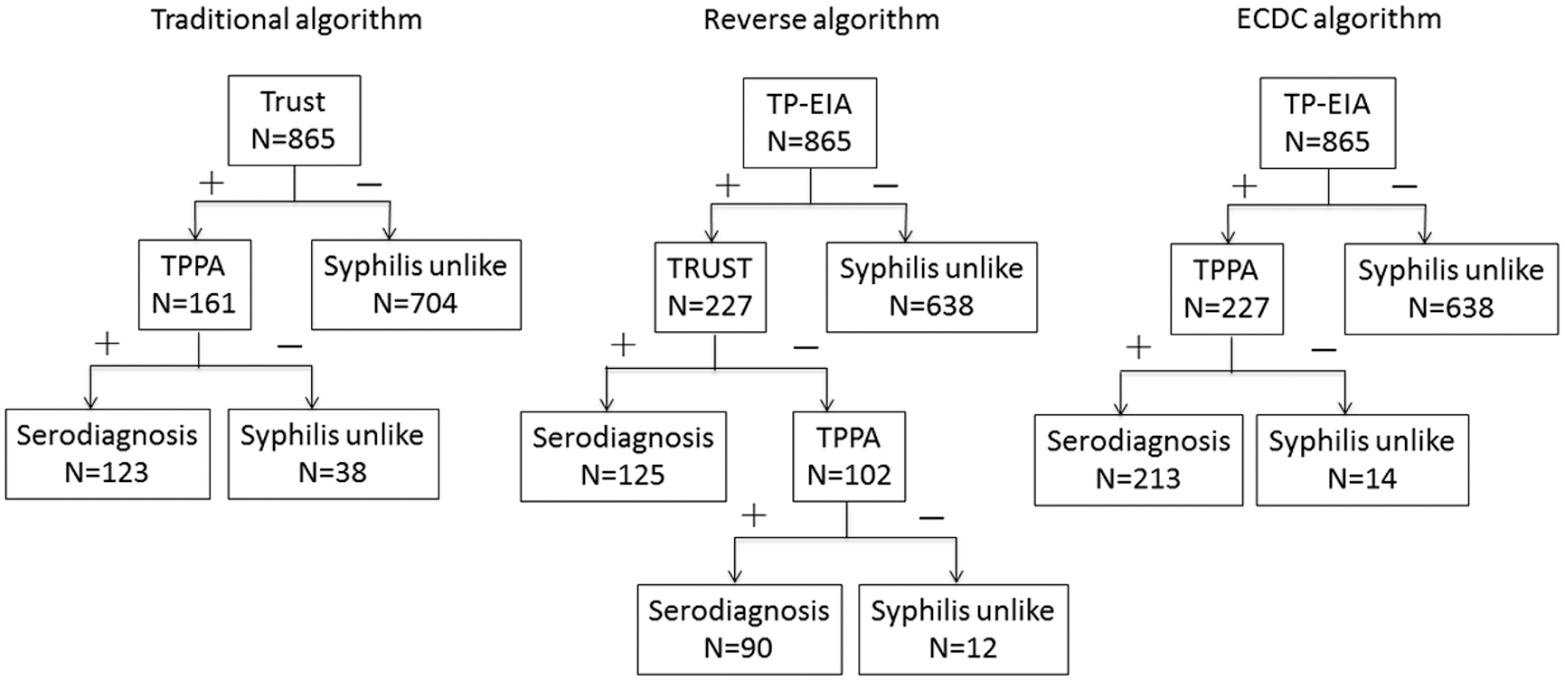
The flow and results of different algorithms. Abbreviations: TRUST, toluidine red unheated serum test; TPPA, *Treponema pallidum* particle agglutination; TP-EIA, *Treponema pallidum* enzyme immunoassay.

The results of syphilis serological testing were interpreted following different algorithms respectively. The definition of serological diagnosis of syphilis under different algorithms is illustrated in Fig 1.

In the traditional algorithm, samples were screened by TRUST test, and the positive samples would be checked by TPPA test. If the TPPA test also gave a positive result, the sample will be considered as positive for syphilis by serodiagnosis.

In the reverse algorithm, samples were screened by TP-EIA test, and the positive samples would be referred to the results of TRUST test. If the TRUST test is positive, the sample is thought to be infected by syphilis. When an inconsistent result was got, the sample would be judged by TPPA test in addition.

In the ECDC algorithm, samples were screened by TP-EIA test, and the reactive samples were confirmed by TPPA test.

### Data analysis

According to the results of TPPA assay, the positive percent agreement, negative percent agreement and total percent agreement, each with 95% confidence interval (CI), of the TP-EIA and TRUST assays were calculated by standard 2 x 2 contingency tables. In addition to percent agreement, kappa coefficients were calculated as a secondary measure of agreement. The seroprevalence of syphilis using traditional and reverse algorithms were compared using McNemar's test for paired proportions. Statistical analysis was performed using SPSS, version 20 (version 20; IBM Corp., Armonk, NY, USA).

## Results

### Demographic characteristics of study participants

As shown in Table 1, the 865 HIV infected individuals had a mean age of 40.7(range 17–81) years, and the male accounted for 82%. The majority of them (87.1%) were of Han ethnicity. More than half (58.7%) of the HIV infected individuals were transmitted by heterosexual. Among the 865 patients, 382(37.9%) patients were in AIDS stage and 1 patient’s stage of HIV infection was unavailable.

**Table 1:** Demographic characteristics of the 865 HIV infected individuals. Serological test results of syphilis.

Characteristic	Subjects No, (%)
Gender	
Male	709 (82%)
Female	156 (18%)
Mean age (years)	40.7±12.3 (range 17–81)
Male	40.2±12.1 (range 17–81)
Female	43.1±12.9 (range 18–75)
Ethnicity	
Han	753 (87.1%)
Other ethnicities	112 (12.9%)
HIV transmission route	
Heterosexual	508 (58.7%)
MSM	225 (26.0%)
Others (injection drug use, blood, unknown)	132 (15.3%)
AIDS stage	
No	536 (62.0%)
Yes	328 (37.9%)
Uncertain	1 (0.1%)

### Serological test results of syphilis

**Fig 2:**
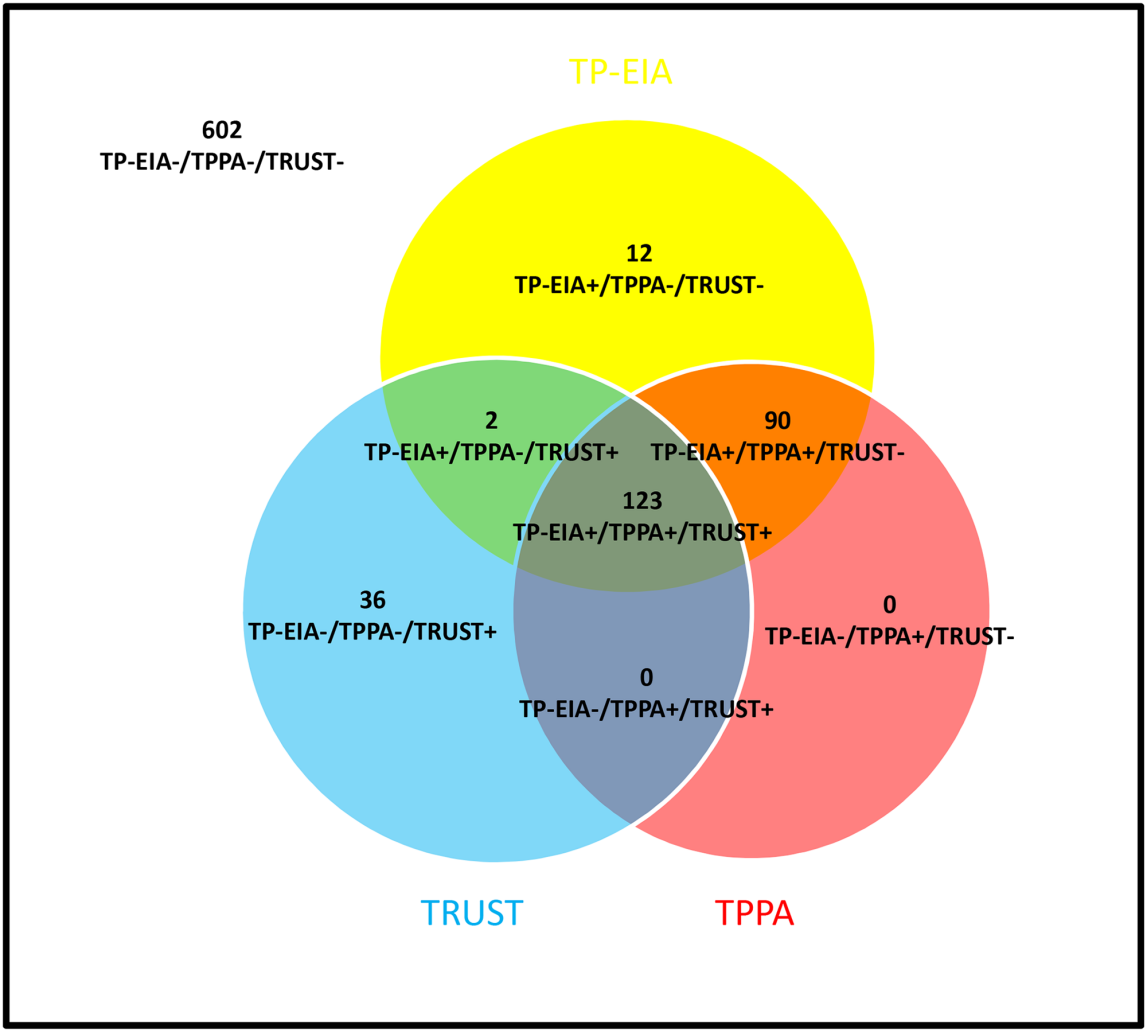
Serological test results of syphilis. Abbreviations: TRUST, toluidine red unheated serum test; TPPA, *Treponema pallidum* particle agglutination; TP-EIA, *Treponema pallidum* enzyme immunoassay.

The serological test results of syphilis are illustrated in Fig 2. Overall, 123 subjects had TP-EIA +/TPPA+/TRUST+ results, and 602 subjects had TP-EIA −/TPPA−/TRUST− results. 90 patients were TP-EIA+/TPPA+/TRUST−. In order to exclude the prozone phenomenon, the TRUST tests for the 90 TP-EIA+/TPPA+/TRUST− subjects were repeated with serum samples diluted from 1:1 to 1:32, and no subjects were found activate with TRUST after dilution.

The total percentages of agreement and corresponding kappa values of each assay’s results compared with those of TPPA were as follows: for TP-EIA, 98.4%, 0.960; for TRUST, 85.2%, 0.566. These data indicated that there was a very good strength of agreement between the TPPA test and the TP-EIA. Using the TPPA test as the standard test, the TP- EIA had 100% positive percent agreement and 97.9% negative percent agreement (Table 2). When the data was analyzed in the AIDS group and non-AIDS group, the results were similar to the total HIV infected individuals (S1 Table).

**Table 2:** Evaluation of TP-EIA and TRUST in comparison with the TPPA assay.

Assay and result	TPPA		% Positive percent agreement (95%CI)	% Negative percent agreement (95%CI)	% total percent agreement (95%CI)	Kappa value (95%CI)
	Positive	Negative				
TP-EIA						
Positive	213	14	100	97.9	98.4	0.960
Negative	0	638	(100–100)	(96.7–99.0)	(97.5–99.2)	(0.937–0.979)
TRUST						
Positive	123	38	57.7	94.2	85.2	0.566
Negative	90	614	(51.1–64.4)	(92.4–96.0)	(82.8–87.6)	(0.493–0.627)

Abbreviations: TRUST, toluidine red unheated serum test; TPPA, *Treponema pallidum* particle agglutination; TP-EIA, *Treponema pallidum* enzyme immunoassay.

### Results of the different algorithms

In the traditional algorithm (Fig 1), 161 (18.6%) samples were reactive with TRUST. Of 161 TRUST positive samples, 123 (76.4%) were confirmed as positive by TPPA and were suggestive of syphilis. 38 (23.6%) were considered to be false-positive by the TRUST. The rate of serodiagnosis of syphilis was 14.2% (95% confidence interval [CI], 11.9%– 16.6%) using the traditional algorithm.

In the reverse algorithm (Fig 1), 227 (26.2%) samples tested positive with TP-EIA. 125 (55.1%) of the 227 TP-EIA positive samples were TRUST-positive. Discordant samples (n = 102) were tested with TPPA and 90 (88.2%) tested positive. 12 (11.8%) samples had negative TPPA results. The rate of serodiagnosis of syphilis was 24.9% (95% CI, 22.0%– 27.7%) using the reverse algorithm.

In the ECDC algorithm (Fig 1), 213 (93.8%) of the 227 TP-EIA positive samples were confirmed by TPPA. The rate of serodiagnosis of syphilis was 24.6% (95% CI, 21.7%– 27.5%) using the ECDC algorithm. Of the 213 samples diagnosed by ECDC algorithm, 123 (57.7%) samples were active with TRUST.

### Comparison of the different algorithms

The reverse algorithm demonstrated significantly higher seroprevalence of syphilis than the traditional algorithm (24.9% vs. 14.2%, *p* < 0.001) in the 865 HIV infected patients. The 123 patients diagnosed by the traditional algorithm were also confirmed by the reverse screening algorithm, while the reverse screening algorithm detected an additional 92 patients that could not be detected using the traditional algorithm. Compared to the reverse algorithm, the traditional algorithm also had a missed serodiagnosis rate of 42.8%. The situation is similarly when compared the traditional algorithm with the ECDC algorithm (with a missed serodiagnosis rate of 42.3%). Among the 92 patients, 90 patients were TP-EIA+/TPPA+/TRUST− and 2 patients were TP-EIA+/TPPA−/TRUST+.

**Fig 3:**
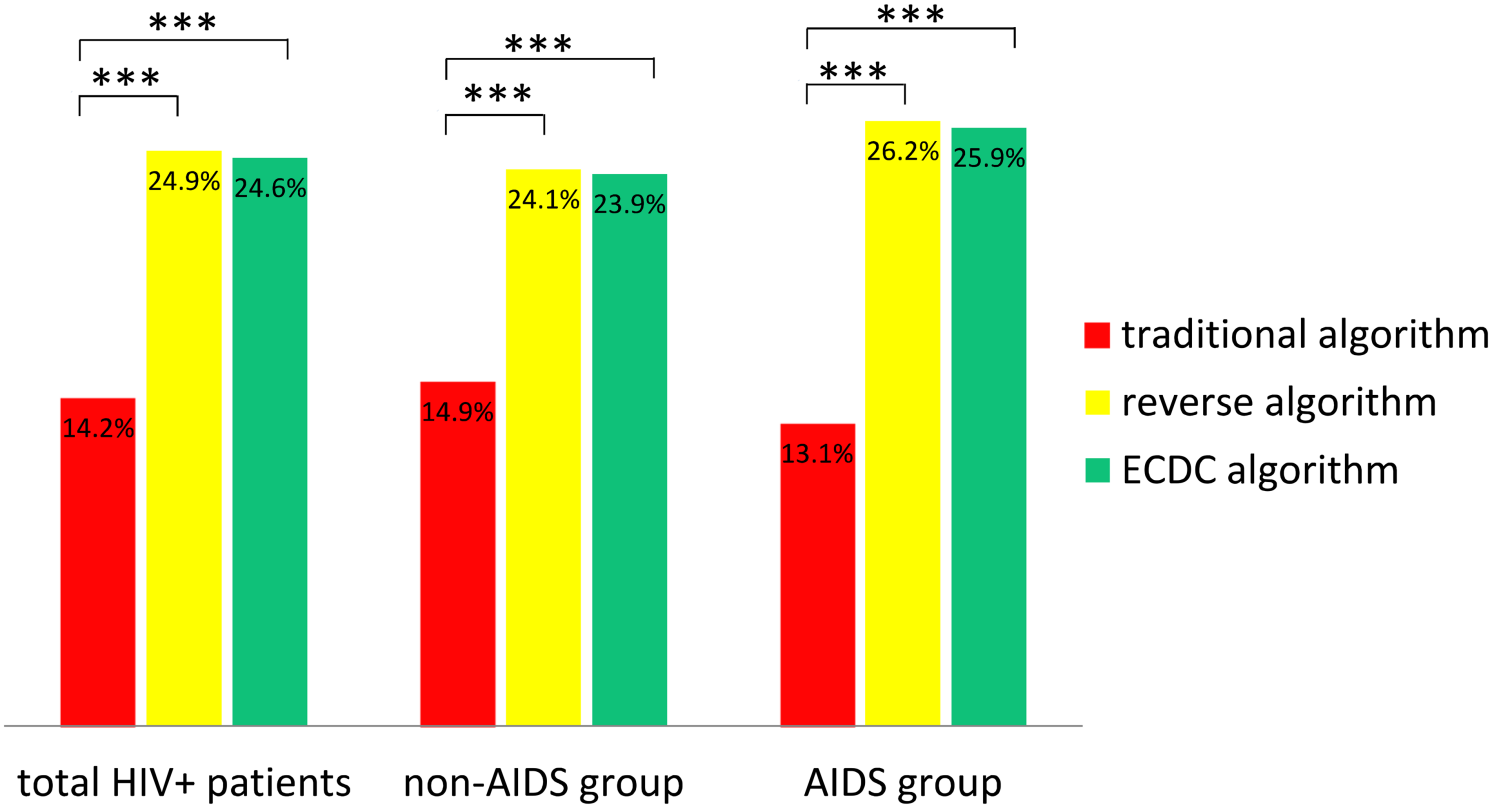
The seroprevalence of syphilis screened by different algorithms. Abbreviations: ECDC, European Centre for Disease Prevention and Control. *** p<0.0001.

The seroprevalence of syphilis screened by traditional algorithm, reverse algorithm and ECDC algorithm in AIDS stage and non-AIDS stage group was 13.1% vs. 14.9% (*p = 0*.*46*), 26.2% vs. 24.1% (*p = 0*.*48*), and 25.9% vs. 23.9% (*p = 0*.*50*), respectively. Both in AIDS stage and non-AIDS stage group, the traditional algorithm showed significantly lower seroprevalence of syphilis than the reverse algorithm and ECDC algorithm (Fig 3).

The total percentages of agreement and corresponding kappa values of each algorithm’s results compared with those of reverse algorithm were as follows: for tradition algorithm, 89.4%, 0.668; for ECDC algirithm,99.8%, 0.994. Using the reverse algorithm as the standard test, the tradition algorithm had 57.2% positive percent agreement and 100% negative percent agreement (Table 3). Compared the traditional algorithm with the reverse algorithm, the positive percent agreement between the non-AIDS group and AIDS group had no statistically significant difference (62% vs. 50%, *p = 0*.*08*, S2 Table).

**Table 3:** Evaluation of traditional algorithm and ECDC algorithm in comparison with reverse algorithm.

Assay and result	Reverse algorithm		% Positive percent agreement (95%CI)	% Negative percent agreement (95%CI)	% total percent agreement (95%CI)	Kappa value (95%CI)
	Positive	Negative				
Traditional algorithm						
Positive	123	0	57.2	100	89.4	0.668
Negative	92	650	(50.5–63.9)	(100–100)	(87.3–91.4)	(0.603–0.729)
ECDC algorithm						
Positive	213	0	99.1	100	99.8	0.994
Negative	2	650	(97.8–100)	(100–100)	(99.4–100)	(0.984–1.0)

Abbreviations: ECDC, European Centre for Disease Prevention and Control.

## Discussion

Serological testing of syphilis remains an important component in the diagnosis of syphilis. Latent syphilis, which is without clinical symptoms, is mainly detected by the non-treponemal and treponemal serologic tests. Treponemal tests become positive in the 2–4 weeks after infection, and it can be detected after successful treatment, even persist lifetime. Non- treponemal tests become positive about 2 weeks later than Treponemal tests. Titers of non-treponemal tests are generally related to disease activity, and it can be declined to negative after successful therapy (except for serofast phenomenon). Non-treponemal tests are mainly used to monitor disease activity and assess the response to treatment. Non-treponemal tests are not sensitive for latent, primary, tertiary syphilis and neurosyphilis, as well as successful treated syphilis. Our study showed a very good strength of agreement between TP-EIA and TPPA, while the TRUST only have a 57.7% positive percent agreement with TPPA(κ = 0.566) in HIV positive patients. These results indicated the insensitive situations of TRUST in HIV infected individuals are common, especially in the AIDS group.

Treponemal tests first or non-treponemal tests first? Does the Order Matter? That is the key difference between the tradition algorithm and the reverse algorithm. Nowadays, there is no uniform screening method for syphilis. Public health decisions on which algorithm should be employed depending on many factors, including disease prevalence, cost, ease of use, and suitability for automation. It is important to consider the screening abilities of different algorithms in the same population. Matthew[7]directly compared the traditional and reverse syphilis screening algorithms in a population with a low prevalence of syphilis. Their results showed that among 1000 patients tested, 6 patients were falsely reactive by reverse screening, compared to none by traditional testing. However, reverse screening identified 2 patients with possible latent syphilis that were missed by traditional testing. In HIV-positive individuals, the situation is more complex. This is the first direct comparison of the reverse and traditional syphilis screening algorithms in a HIV-infected population. Our present study found that among 865 patients tested, the reverse screening algorithm diagnosed an additional 92 patients that could not be observed using the traditional algorithm. The missed diagnosis rate of the traditional screening algorithm was 42.8% compared with the reverse screening algorithm, which is higher than the study by Tong [8]. That was a large survey conducted in an area with a high prevalence of syphilis (11.4%). Previous studies[9] have suggested that reverse screening can yield a high false-positive rate, while many early studies lacked parallel traditional screening on the same samples. Our study found the false-positive rate of reverse screening was lower than traditional screening (1.4% vs. 4.4%) and our finding were consistent with Tong’s findings. The prevalence of syphilis of the participants may contribute to the difference. We and Tong’s study were carried out in a population with a high prevalence of syphilis.

Our study showed there was a very good strength of agreement between the reverse and ECDC algorithm, and demonstrated that the seroprevalence of syphilis using the reverse algorithm (or the ECDC algorithm) was significantly higher than the traditional algorithm for HIV-positive individuals. The 92 patients missed by traditional algorithm contribute to this difference, of which, 90 TRUST−/TP-EIA+/TPPA+ patients were the majority. Patients with discordant TRUST and TP-EIA serological results are confirmed by TPPA. If TPPA is non-reactive, it is considered to be false-positive. When TPPA is reactive, there are 3 interpretations (i) successfully treated syphilis infection; (ii) early/late or latent syphilis, when the sensitivity of TRUST is low; (iii) the prozone phenomenon, especially in secondary syphilis. The prozone phenomenon in syphilis testing refers to a false-negative response resulting from an excess of antibody, which prevents visible agglutination in agglutination or precipitation tests. Beyond our expectation, no prozone phenomenon were found among the 90 TRUST−/TP-EIA+/TPPA+ patients in the present study, which is lower than Jeffrey’s [10]study (0.90%, 2/223). May be it is due to the small sample size, and it needs to evaluate the rate of prozone phenomenon in HIV infected individuals in a larger sample size.

There are several limitations to our study and the results should be interpreted with caution. First, all specimens were obtained from hospital patients and there is consequent sample selection bias. Second, the study was conducted from the perspective of serological diagnosis, and both the clinical diagnosis and prior history of syphilis were not analyzed.

In conclusion, screening of HIV-populations using different algorithms may result in a statistically different seroprevalence of syphilis. When comparing the prevalence of syphilis in HIV-infected individuals from different surveys, it is important to assess which screening method is employed. Finally, we advocate the reverse algorithm (or the ECDC algorithm) approach for the screening of syphilis in HIV-infected populations, given its sensitivity for early/late and latent syphilis. The quantitative non-treponemal tests were recommended to determine serological activity of syphilis in ECDC algorithm. The tradition algorithm approach underestimates the prevalence of syphilis in HIV-infected individuals.

## Supporting information

S1 Table Evaluation of TP-EIA and TRUST in comparison with the TPPA assay in non-AIDS group and AIDS group. Abbreviations: TRUST, toluidine red unheated serum test; TPPA, *Treponema pallidum* particle agglutination; TP-EIA, *Treponema pallidum* enzyme immunoassay.
(DOCX)

S2 Table Evaluation of traditional algorithm and ECDC algorithm in comparison with reverse algorithm in non-AIDS group and AIDS group. Abbreviations: ECDC, European Centre for Disease Prevention and Control.
(DOCX)

S1 File Raw data of the HIV-infected individuals. (XLSX)
