# Supplementary material for: The tradition algorithm approach underestimates the prevalence of serodiagnosis of syphilis in HIV-infected individuals
Source: PLoS Negl Trop Dis. 2017 Jul 20;11(7):e0005758. doi: 10.1371/journal.pntd.0005758 (PMC5538742; doi:10.1371/journal.pntd.0005758)
Supplement: S1 Table — Abbreviations: TRUST, toluidine red unheated serum test; TPPA, Treponema pallidum particle agglutination; TP-EIA, Treponema pallidum enzyme immunoassay. (DOCX) [file pntd.0005758.s001.docx]

| Assay and result | TPPA | | % Positive percent agreement (95%CI) | % Negative percent agreement (95%CI) | % total percent  agreement (95%CI) | Kappa value  (95%CI) |
| --- | --- | --- | --- | --- | --- | --- |
|  | Positive | Negative |  |  |  |  |
| Non-AIDS group | | | | | | |
| TP-EIA | | | | | | |
| Positive | 128 | 8 | 100 | 98.0 | 98.5 | 0.96 |
| Negative | 0 | 400 | (100- 100) | (96.7-99.4) | (97.5-99.5) | (0.928-0.984) |
| TRUST | | | | | | |
| Positive | 80 | 23 | 62.5 | 94.4 | 86.8 | 0.609 |
| Negative | 48 | 385 | (54.0-71.0) | (92.1-96.6) | (83.9-89.6) | (0.515-0.681) |
| AIDS group | | | | | | |
| TP-EIA | | | | | | |
| Positive | 85 | 6 | 100 | 97.5 | 98.2 | 0.953 |
| Negative | 0 | 237 | (100-100) | (95.6-99.5) | (96.7-99.6) | (0.913-0.985) |
| TRUST | | | | | | |
| Positive | 43 | 15 | 50.6 | 93.8 | 82.6 | 0.495 |
| Negative | 42 | 228 | (39.7-61.4) | (90.8-96.9) | (78.5-86.7) | (0.367-0.598) |
