# Supplementary material for: The tradition algorithm approach underestimates the prevalence of serodiagnosis of syphilis in HIV-infected individuals
Source: PLoS Negl Trop Dis. 2017 Jul 20;11(7):e0005758. doi: 10.1371/journal.pntd.0005758 (PMC5538742; doi:10.1371/journal.pntd.0005758)
Supplement: S2 Table — Abbreviations: ECDC, European Centre for Disease Prevention and Control. (DOCX) [file pntd.0005758.s002.docx]

| Assay and result | Reverse algorithm | | % Positive percent agreement (95%CI) | | % Negative percent agreement (95%CI) | % total percent  agreement (95%CI) | Kappa value  (95%CI) |
| --- | --- | --- | --- | --- | --- | --- | --- |
|  | Positive | Negative |  |  |  |  |  |
| Non-AIDS group | | | | | | | |
| Traditional algorithm | | | | | | | |
| Positive | 80 | 0 | | 62.0 | 100 | 90.9 | 0.713 |
| Negative | 49 | 407 | | (53.5-70.5) | (100- 100) | (88.4-93.3) | (0.627-0.783) |
| ECDC algorithm | | | | | | | |
| Positive | 128 | 0 | | 99.2 | 100 | 99.8 | 0.995 |
| Negative | 1 | 407 | | (97.7-100) | (100- 100) | (99.4-100) | (0.983-1.0) |
| AIDS group | | | | | | | |
| Traditional algorithm | | | | | | | |
| Positive | 43 | 0 | | 50.0 | 100 | 86.9 | 0.596 |
| Negative | 43 | 242 | | (39.2-60.8) | (100- 100) | (83.2-90.6) | (0.476-0.693) |
| ECDC algorithm | | | | | | | |
| Positive | 85 | 0 | | 98.8 | 100 | 99.7 | 0.992 |
| Negative | 1 | 242 | | (96.5-100) | (100- 100) | (99.1-100) | (0.974-1.0) |
